# The Regeneration of Large-Sized and Vascularized Adipose Tissue Using a Tailored Elastic Scaffold and dECM Hydrogels

**DOI:** 10.3390/ijms222212560

**Published:** 2021-11-22

**Authors:** Su Hee Kim, Donghak Kim, Misun Cha, Soo Hyun Kim, Youngmee Jung

**Affiliations:** 1 Biomaterials Research Center, Korea Institute of Science and Technology (KIST), 5, Hwarang-ro 14-gil, Seongbuk-gu, Seoul 02792, Korea; sweess@imedifab.com (S.H.K.); cherish7693@korea.ac.kr (D.K.); 2 R&D Center, Medifab Co., Ltd., 70 Dusan-ro, Geumcheon-gu, Seoul 08584, Korea; cmsbest@imedifab.com; 3 KU-KIST Graduate School of Converging Science and Technology, Korea University, 145 Anam-ro, Seongbuk-gu, Seoul 02841, Korea; 4 Korea Institute of Science and Technology (KIST) Europe, Campus E 7.1, 66123 Saarbrücken, Germany; 5 School of Electrical and Electronic Engineering, YU-KIST Institute, Yonsei University, Seoul 03722, Korea

**Keywords:** adipose tissue regeneration, decellularized extracellular matrix (dECM) hydrogel, patient-specific elastic scaffolds, angiogenesis, tissue engineering

## Abstract

A dome-shaped elastic poly(l-lactide-*co*-caprolactone) (PLCL) scaffold with a channel and pore structure was fabricated by a combinative method of 3D printing technology and the gel pressing method (13 mm in diameter and 6.5 mm in thickness) for patient-specific regeneration. The PLCL scaffold was combined with adipose decellularized extracellular matrix (adECM) and heart decellularized extracellular matrix (hdECM) hydrogels and human adipose-derived stem cells (hADSCs) to promote adipogenesis and angiogenesis. These scaffolds had mechanical properties similar to those of native adipose tissue for improved tissue regeneration. The results of the in vitro real-time PCR showed that the dECM hydrogel mixture induces adipogenesis. In addition, the in vivo study at 12 weeks demonstrated that the tissue-engineered PLCL scaffolds containing the hydrogel mixture (hdECM/adECM (80:20)) and hADSCs promoted angiogenesis and adipose tissue formation, and suppressed apoptosis. Therefore, we expect that our constructs will be clinically applicable as material for the regeneration of patient-specific large-sized adipose tissue.

## 1. Introduction

Severe adipose and soft tissue defects, due to the resection of breast tumors or due to accidents, are associated with not only cosmetic issues but also the mental suffering of the patient. In this regard, the regeneration of adipose tissue is clinically important [1,2,3]. Various treatments for regenerating adipose tissue have been performed in clinical practice. Clinical approaches use autologous flaps, enriched grafts, liposuctioned tissue, and allogenic or synthetic fillers, such as fibrin, hyaluronic acid, and viscoelastic hylan gels [4,5,6]. Despite improved surgical techniques, these treatments encompass substantial donor site morbidity and often yield limited success in terms of integration and the maintenance of volume and shape, calling for alternatives to current reconstructive procedures.

Many tissue engineering approaches using various biomaterials and cells have been studied [2,7,8,9,10,11]. Numerous studies have been conducted to improve the efficiency of tissue regeneration by regulating cellular behaviors using materials capable of mimicking the extracellular matrix of native tissue. In recent years, research on tissue regeneration using patient-specific 3D printing technology has been actively carried out. The development of materials and 3D printing processes for regenerating adipose or soft tissues has been published [12,13,14,15,16,17,18].

Hong et al. developed a highly stretchable and tough hydrogel for 3D printing [14]. The hydrogel is a mixture of poly(ethylene glycol) diacrylate and alginate and has stretchable and tough mechanical properties, such as covalent crosslinking by UV, and ionic crosslinking by calcium ions. The authors reported that when the hydrogels and cells were bioprinted, the cell viability and recovery rate after strain were excellent.

Wu et al. reported cell-laden tissue constructs by bioprinting with hydrogels and cells [19]. A mixture of gelatin, alginate, and collagen was printed with cells, and it had controllable degradation. They stated that the printed cells in the 3D constructs grew faster, had a much better capacity to proliferate, and expressed greater specific marker proteins, indicating that this work may help to improve the alginate bioink system for the application of 3D printing in tissue engineering.

In addition to studies using hydrogels, dual materials of biodegradable polymers and hydrogels have also been used to reinforce mechanical properties [20]. Hyun-Wook Kang et al. studied a 3D bioprinting system to produce human-scale tissue constructs [21]. They used polycaprolactone (PCL), a hydrogel mixture of gelatin, fibrinogen, and hyaluronic acid, as well as myoblasts for the regeneration of skeletal muscle. The constructs consisted of a PCL pillar and a cell-laden hydrogel pattern, confirming that tissue regeneration was successful when transplanted subcutaneously into nude rats. Falguni Pati et al. reported biomimetic 3D tissue printing for adipose tissue regeneration [22]. They used PCL as a structural support and an adipose decellularized extracellular matrix (adECM) hydrogel as a bioactive material and as a human adipose-derived stem cell (hADSC) delivery system. The authors concluded that the PCL/adECM hydrogel/hADSC constructs that they developed were superior to the injectable decellularized extracellular matrix (dECM) hydrogel/hADSCs in terms of cell survival and volume maintenance.

The above studies have developed 3D printing materials and processes for soft tissue regeneration and have shown excellent tissue regeneration abilities. However, there are some limitations to their use in large-sized adipose tissue regeneration. In the case of materials that use hydrogels, the mechanical properties are so low that the implanted constructs may not survive at the implantation site until mature tissue is formed in the body. In addition, the crosslinking of covalent bonds for the improvement of mechanical properties may have a residual problem in the body, unlike biodegradable biomaterials. PCL is a biodegradable polymer that is widely used in 3D-printed implants. However, this material may have limitations in terms of tissue regeneration abilities, foreign body sensations, and engraftment rates because its mechanical properties do not match those of native adipose tissue [22]. Poly(l-lactide-*co*-ε-caprolactone) (PLCL), a biodegradable and biocompatible polymer, is a flexible and rubber-like elastic random copolymer [23]. PLCL has been widely applied in soft tissue engineering such as adipose tissues, blood vessels, and skin, because it has suitable mechanical properties for soft tissue regeneration [24,25]. Furthermore, it can overcome the limitation of hydrogels and PCL and provide a 3D spatial structure.

In this study, we aimed to develop a method for regenerating patient-specific large-sized adipose tissue and evaluate its efficacy. To achieve this, we tried to overcome the existing limitations and applied 3D printing technology to PLCL, a highly elastic biodegradable polymer. We also introduced factors to regenerate large adipose tissue. By using a water-soluble 3D printing mold, mass transfer to the scaffold was achieved by creating a channel structure inside the scaffold, and the effects of angiogenesis and adipogenesis were increased by using the heart and adipose dECM hydrogels. The developed constructs were evaluated for efficacy and tissue regeneration abilities through various in vitro and in vivo evaluations.

## 2. Results

### 2.1. The Characterization of a PLCL Scaffold

**Figure 1:**
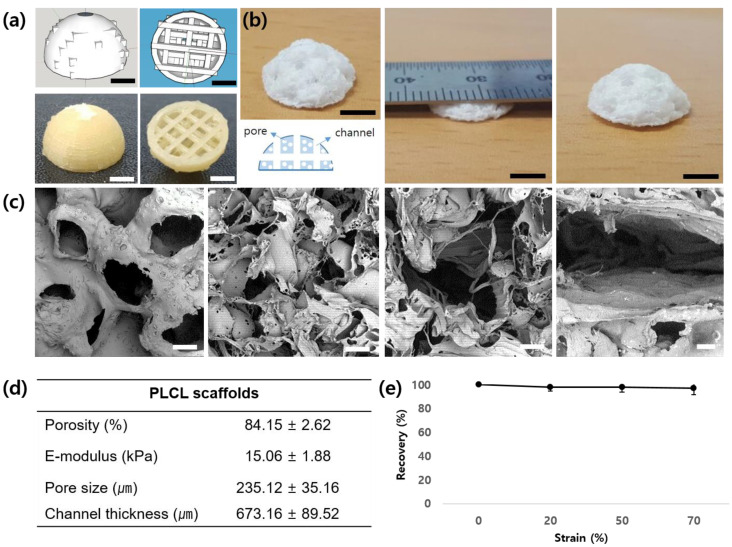
Characterization of a patient-specific PLCL scaffold. (**a**) Design for a PVA mold and 3D-printed PVA mold; (**b**) PLCL scaffold that has channels and a porous structure (scale bar: 5 mm); (**c**) SEM images of the scaffold (scale bar: 100 μm); (**d**) Characteristics of the scaffold; (**e**) Recovery after compressive strain.

We developed a patient-specific scaffold for large soft tissue regeneration. A dome-shaped elastic PLCL scaffold with a channel and pore structure was fabricated using a combination of 3D printing technology and a gel pressing method. Three-dimensional printing was used to create a sacrificial PVA mold for the factorial design of the scaffold and the inner channel structure. The pore structure was produced by injecting PLCL/NaCl particles/THF paste into the PVA mold and removing the solvent and NaCl particles. Finally, a highly pore-interconnected and tailored scaffold was created. It had a diameter of 13 mm, a thickness of 6.5 mm, and a degree of size congruity of 93.25% when compared with the PVA mold. The morphology of the scaffold was observed using SEM (Figure 1). Many open pores were created on the surface and in the inner part of the scaffold. Channels were built in a porous structure and were connected to the edge of the scaffold. As shown in Figure 1b, PLCL scaffolds can be divided into quadrants along three imaginary longitudinal axes; the two central compartments are expressed as the “center”, and the two side compartments are expressed as the “side”. The mean pore size was 235.12 ± 35.16 μm and the thickness of the channel was 673.16 ± 89.52 μm. The porosity of the scaffold was 84.15 ± 2.62%. The mechanical properties were measured using a universal test machine. The compressive modulus of the scaffold was 15.06 ± 1.88 kPa and 97.30% recovery appeared after 70% strain.

### 2.2. The Characterization of Decellularized Adipose and Heart dECMs

**Figure 2:**
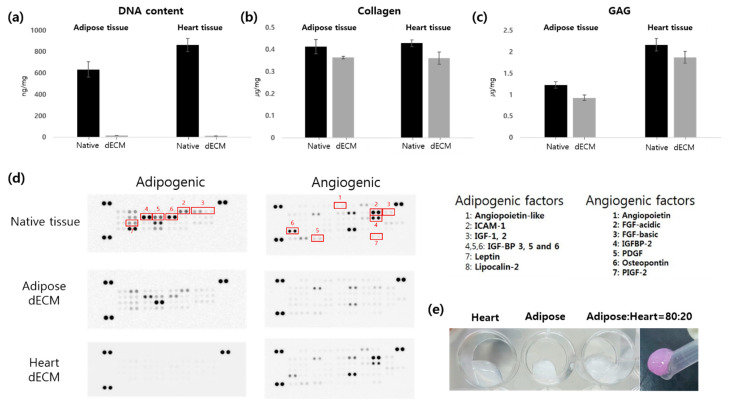
Characterization of adipose and heart dECM. (**a**) DNA; (**b**) collagen; and (**c**) GAG contents of native tissues, adipose dECM, and heart dECM. (**d**) Analysis of cytokines in the native tissues, adipose dECM, and heart dECM. (**e**) Appearance of the dECM hydrogels.

Adipose tissue- and heart tissue-derived dECM-based hydrogels were used to enhance adipogenic differentiation and angiogenesis. First, residual DNA, collagen, and the GAG contents of dECM were investigated. When the DNA content of dECM was compared with native tissues, 97.26% and 98.50% of the DNA was removed from the adipose and heart tissues, respectively. In contrast, 88.13% and 84.21% of collagen remained in the adipose and heart dECMs, respectively. Moreover, 75.61% and 86.63% of GAGs were present in the adipose and heart dECMs, respectively (Figure 2).

Adipose tissue contains many kinds of cytokines for adipogenesis, such as insulin growth factors-1 and -2, as well as lipocalin-2. Heart tissue has various angiogenic factors, such as angiopoietin and the platelet-derived growth factor. We determined these cytokines in the dECM using a microarray kit. A large amount of soluble cytokines were washed out during the decellularization process. However, some cytokines trapped in the dECM matrix remained. In particular, key adipogenic and angiogenic factors existed in the dECM, and their bioactivity was evaluated in vitro and in vivo.

### 2.3. In Vitro Adipogenic Gene Expression (Real-Time PCR)

**Figure 3:**
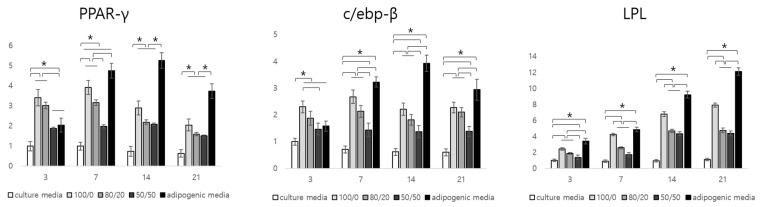
Relative gene expression of the mRNA of PPAR-, c/ebp-, and LPL of hADSCs/constructs culture in vitro for up to 21 days (* *p* < 0.05).

We confirmed the presence of residual cytokines in the adipose dECM using a microarray. In this study, we investigated the influence of adipogenic factors on the adipogenesis of hADSCs in vitro. We compared the expression of five groups (Group 1: scaffold + hADSCs in CM; Group 2: scaffold + adipose dECM hydrogel + hADSCs in CM; Group 3: scaffold + adipose dECM/heart dECM (80/20) + hADSCs in CM; Group 4: scaffold + adipose dECM/heart dECM (50/50) + hADSCs in CM; Group 5: scaffold + hADSCs in AM). The relative gene expression of PPAR-γ and c/ebp-β as initial signaling and LPL as downstream signaling during adipogenic differentiation was determined (Figure 3).

According to the results, the gene expression of PPAR-γ and c/ebp-β in Group 2 (adipose dECM gel only) was the highest among the groups at day 3. The PPAR-γ gene level in Group 2 was about 3.42 and 1.81 times higher than those of Groups 1 and 4, respectively. This value was also approximately 1.68 times higher than that of the positive group at the initial stage of differentiation. The gene level of Group 3 was lower than that of Group 2. However, there were no significant differences between groups. The c/ebp-β gene expression was similar to that of the PPAR-γ gene expression. The c/ebp-β gene expression in Group 2 was about 2.30 and 1.45 times higher than that of the negative and positive controls, respectively. Although both PPAR-γ and c/ebp-β were more highly expressed in Groups 2 and 3 compared to the negative control, they had lower values compared to the positive group from day 7 to 21. There were no additional adipogenic factors in the samples of Groups 2, 3, and 4. Gene expressions in Groups 2, 3, and 4 containing the dECM hydrogel increased by day 7 then subsequently decreased by day 21. The gene expression of the positive control increased by day 14 and then decreased at day 21. PPAR-γ and c/ebp-β genes were considered to be related to the initial stage of adipogenic differentiation. On the other hand, the gene expression of LPL in Group 2 was about 2.44 times higher than that of the negative control and about 1.39 times lower than that of the positive control. When it was compared with Groups 3 and 4 containing adipose dECM gel, gene expressions of about 1.29 and 1.77 times higher were observed in Group 2, respectively. The level of LPL gene expression increased steadily by day 21, which is why LPL is involved in the late stage of adipogenic differentiation.

### 2.4. Angiogenesis and Apoptosis of In Vivo Samples

**Figure 4:**
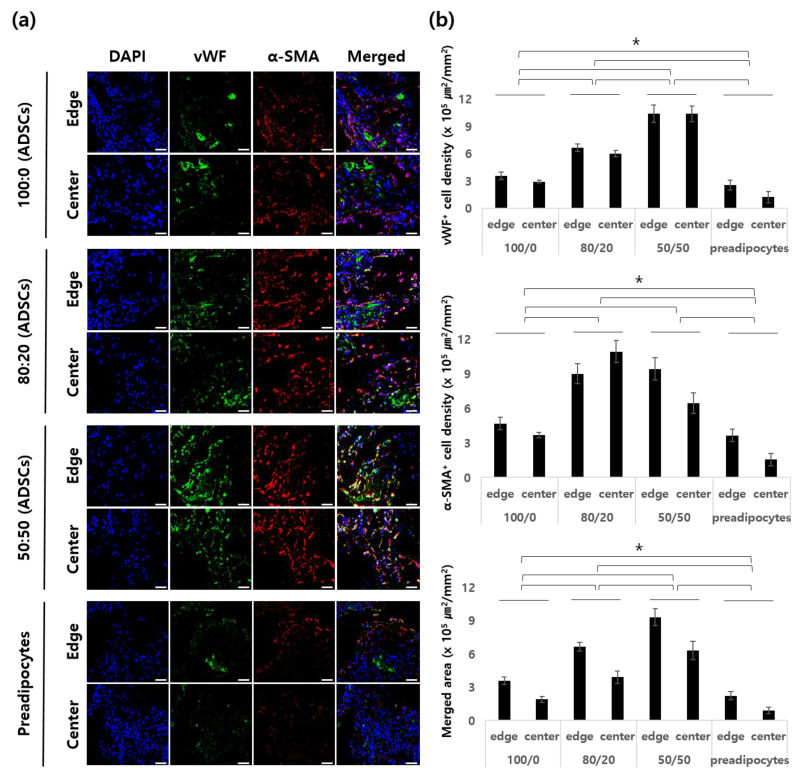
Angiogenesis in a construct. (**a**) Representative immunostaining of endothelial cells (ECs) and smooth muscle cells (SMCs) in each group 1 week after implantation. (**b**) Quantification of the vWF^+^ cell density, α-SMA^+^ vessel density, and merged area of vWF^+^ and α-SMA^+^ vessels (μm^2^/mm^2^). Scale bar: 100 μm (* *p* < 0.05).

In this study, we investigated the angiogenesis and apoptosis of seeded hADSCs. Angiogenesis at the edge and the center of the scaffolds was observed by vWF and α-SMA staining (Figure 4). We hypothesized that angiogenic factors contained in the heart dECM hydrogel induced more blood vessels inside the scaffolds. To evaluate this, we investigated the angiogenic effect of heart dECM hydrogel by the ratio of heart dECM hydrogel to the whole dECM hydrogel. The results of 1 week showed that the stained area of vWF^+^ cells of Group 3, which included adECM/hdECM (50:50), was the largest, followed by Group 2, which included hdECM/adECM (80:20). Group 1, which included only the adECM hydrogel, had a smaller stained area among the groups containing dECM hydrogels; however, it had a larger stained area than Group 4, which included only cells. In the case of α-SMA^+^ cells, the stained areas of Groups 2 and 3 were similar at the edges. On the other hand, the stained area of Group 2 was higher than that of Group 3 at the center. The maturation index was similar to that of vWF^+^ staining. We could observe not only the amount of blood vessels but also their size. Groups 2 and 3, which contained the hdECM hydrogel, had larger blood vessels than Groups 1 and 4. From these results, we concluded that the hdECM hydrogel induced more and larger blood vessels in the in vivo environment.

**Figure 5:**
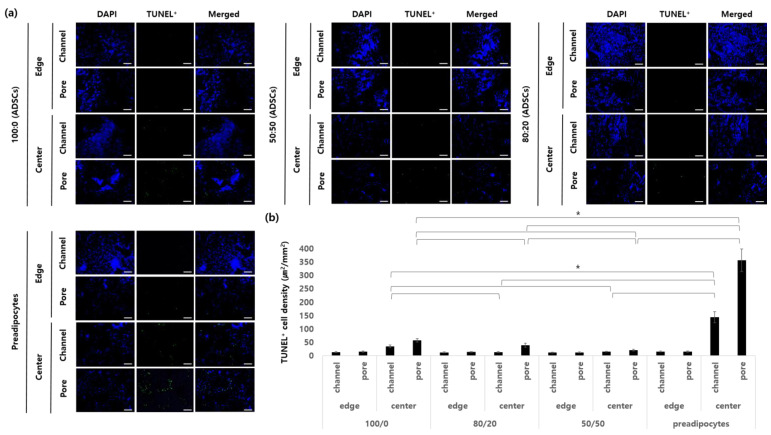
Apoptosis of seeded cells in a construct. (**a**) Representative immunostaining of apoptotic cells. (**b**) Quantification of the stained area (μm^2^/mm^2^). Scale bar: 100 μm (* *p* < 0.05).

The apoptosis of hADSCs was observed 1 week after implantation. The section of the sample was separately observed in four parts according to the position (Figure 5). We hypothesized that the inner channel structure and newly formed blood vessels by angiogenic factors prevented hypoxia in cells and prevented their apoptosis. At the edges of all the groups of 1-week samples, apoptotic cells were rarely observed not only in the channel structure but also in the porous structure. On the other hand, there were significant differences in the central parts of all samples. Groups 2 and 3 had the lowest degree of the apoptosis of hADSCs because more and larger blood vessels were induced compared to the other groups. In Group 1, which contained only adipose dECM hydrogel, there were lower numbers of apoptotic cells in the channel area; however, more cells died in the porous region of the center than in Groups 2 or 3. Among the groups, the largest number of apoptotic cells was observed in Group 4, which contained only preadipocytes. In particular, in the porous region of the center part, about 10.4 times more cells died compared to Group 2. This phenomenon was considered to be caused by a lack of blood vessel formation.

We observed the blood vessels and apoptosis of the cells harvested between 4 and 12 weeks after their implantation in subcutaneous nude mice (Supplementary Figures S1 and S2). The blood vessels were mature blood vessels in all groups compared to the first week, and larger amounts of blood vessels were still forming in Groups 2 and 3. However, the apoptosis of cells was significantly reduced in all groups. Although Groups 1 and 4 showed less angiogenesis than Groups 2 and 3, apoptosis in the early stage of implantation was stopped due to the formation of blood vessels for 12 weeks.

### 2.5. The Histological and Immunofluorescence Staining of In Vivo Samples

**Figure 6:**
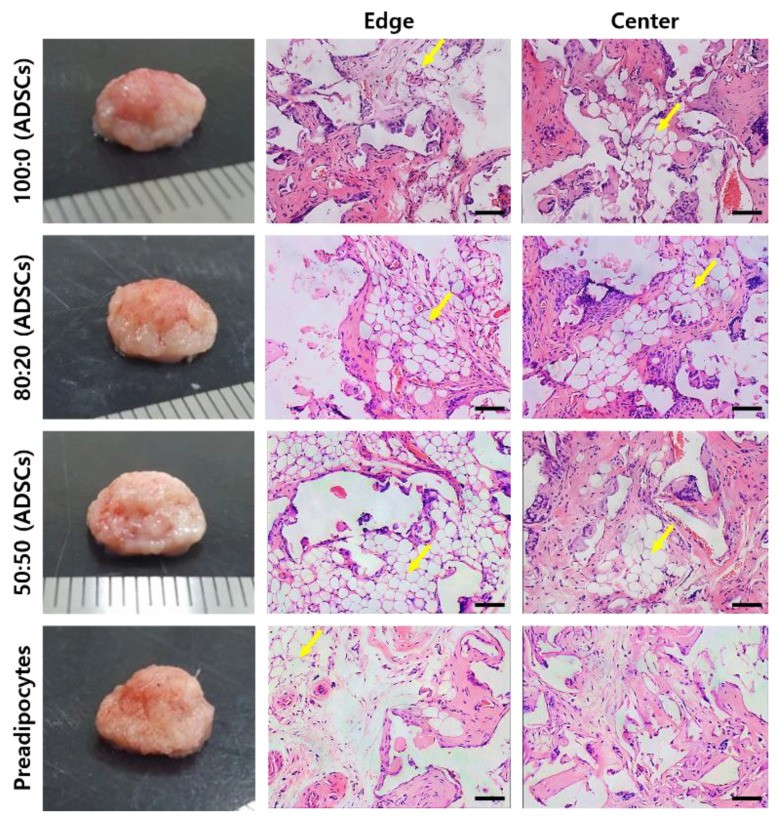
H&E staining for each group of the 12-week in vivo samples (yellow arrows: mature adipose tissue). Scale bar: 100 μm.

We performed a histological analysis of the in vivo samples of 12-week samples by H&E staining. In Figure 6, mature adipose tissue (yellow arrows) was observed at the edge and center parts of the three groups containing dECM hydrogels. Additionally, blood vessels were induced and retained inside the tissue, even at the center. On the other hand, mature adipose tissue was formed only at the edge of the group containing only preadipocytes. Immature morphology was observed at the center of the scaffold.

**Figure 7:**
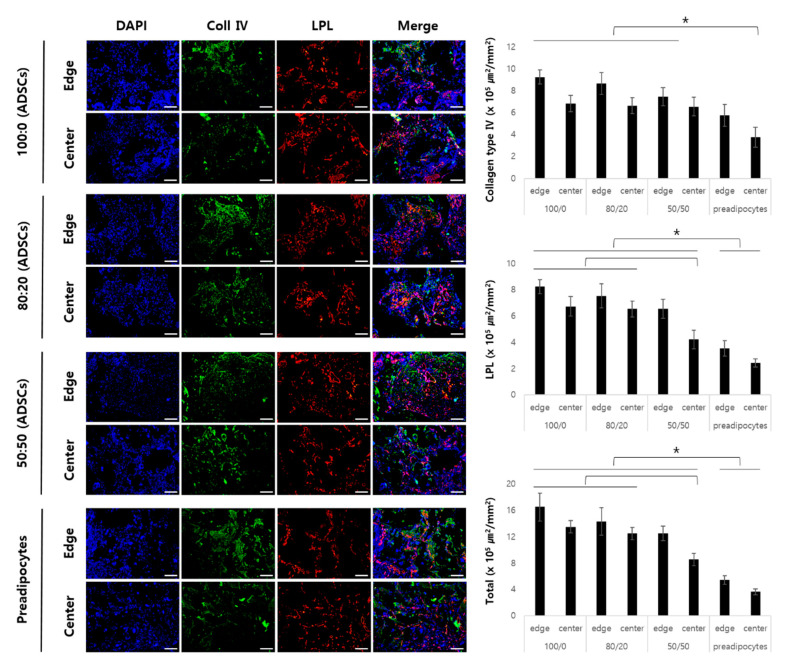
Immunofluorescence staining adipogenic markers for each group of the 12-week in vivo samples. Scale bar: 100 μm (* *p* < 0.05).

Figure 7 shows the expression of type IV collagen with similar average values at the edges and centers of Groups 1, 2, and 3. On the other hand, in the center of Group 4, a significantly lower expression of type IV collagen was observed when compared to that of the other groups.

The stained area of LPL had similar average values in Groups 1 and 2, but the center of Group 3 showed a low stained area when compared with that of Groups 1 and 2. In addition, LPL expression in Group 4 was significantly lower than that in Groups 1, 2, and 3.

### 2.6. Mechanical Properties and Accumulative Lipids of In Vivo Samples

From a clinical point of view, mechanical properties such as elasticity and the e-modulus are important factors in the regeneration of soft tissue. In this study, we used an elastic polymer to mimic the mechanical properties of the soft tissue. Matching the mechanical properties of implants with native tissue is important for tissue regeneration.

**Figure 8:**
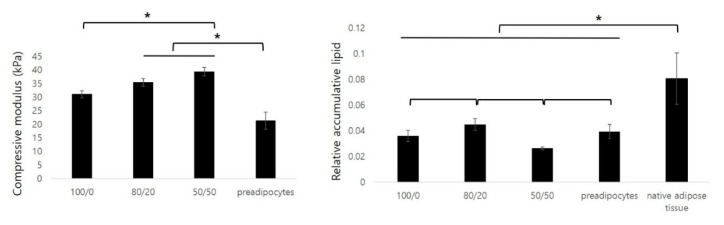
Compressive modulus and relative accumulative lipids for each group of the 12-week in vivo samples (* *p* < 0.05).

The compressive moduli of the in vivo samples were investigated using UTM (Figure 8). The three groups containing dECM gels and hADSCs had comparatively higher values of the e-modulus compared with those of Group 4, containing only preadipocytes. Groups 1, 2, 3, and 4 had 31.04 ± 1.36, 35.44 ± 1.39, 39.59 ± 1.50, and 21.33 ± 3.13 kPa, respectively. 

The adipose tissue regulates lipid metabolism. Mature adipocytes secrete lipids to store energy. In this study, we determined lipid accumulation in newly formed tissues. The largest amount of lipids accumulated in the constructs of Group 2. The amount of lipids was then increased in the order of Groups 1, 4, and 3. Compared with the lipid amount of native adipose tissue, Group 1 was 39.77 ± 2.18%, Group 2 was 43.61 ± 1.32%, Group 3 was 32.67 ± 3.10%, and Group 4 was 33.51 ± 4.06%.

## 3. Discussion

The regeneration of large-sized adipose tissue is very important in terms of plastic surgery after the resection of tumors or cosmetic surgery. In the present study, we considered three important factors for the successful regeneration of large adipose tissue: (1) the fabrication of patient-specific and elastic scaffolds; (2) the enhancement of mass transfer into the scaffolds and angiogenesis for cell survival; and (3) the adipogenic differentiation of seeded hADSCs.

In this study, we mimicked not only the patient-specific shapes but also the mechanical properties of adipose tissue. The value of the e-modulus of the scaffold was 15.06 ± 1.88 kPa and 97.30% recovery appeared after 70% strain. According to the reference, adipose tissue has a Young’s modulus of 17–23 kPa with a standard variation of 4 kPa [26]. Compared with this reference, our scaffold has mechanical properties similar to those of native tissue. The elasticity and e-modulus values were controlled by the elasticity of the material and the microstructure of the scaffold. Ideally, the scaffold should have mechanical properties consistent with the anatomical site into which it is to be implanted, and from a practical perspective, it must be strong enough to allow surgical handling during implantation.

A highly porous scaffold with a channel and pore structure was fabricated using an indirect 3D printing method. Mass transfer is a critical factor for successful tissue regeneration in large-sized tissue regeneration based on a scaffold [27]. The improvement in the viability of delivered cells is a critical factor for successful cell therapy. The supply of adequate oxygen and nutrients by diffusion affects cell viability prior to the formation of blood vessels in the scaffold. For this reason, this study intends to implement a structure that can be well-diffused. The inner channel structure and highly porous architecture enhances mass transport and provides implanted cells with a good environment. In Figure 4, 1 week after implantation, Group 4, in which mature blood vessels were hardly formed at the center of the scaffold, showed that apoptosis of seeded cells was more frequent than in other groups, but only a few cells died. In particular, the cells near the center channel died less than those near the center pores. This suggests that the channel structure has a favorable effect on mass transport, which may result in decreased cell death. Durham et al. manufactured and evaluated channeled scaffolds composed of poly(l-lactic acid) in vitro. They reported that channels in thick scaffolds promoted cell penetration and tissue formation by the mass transfer of nutrients to the scaffold interior [28].

It was demonstrated by Jeong et al. that highly porous scaffolds have slow degradation properties in vivo. [29] The molecular weight of PLCL scaffolds decreased gradually to 81% during 15 weeks of subcutaneous implantation in nude mice. The MW was 76% of the initial value after 3 weeks and 39% after 15 weeks. An in vitro degradation analysis showed that the mass of PLCL scaffolds decreased slowly to 90% after 26 weeks and to 50% at 52 weeks. [30]

Angiogenesis is critical for the successful regeneration of large-sized tissue. Sufficient blood vessels prevent hypoxia and apoptosis, which can lead to the proliferation and differentiation of cells over a long period [31,32]. In particular, the regeneration of vascularized adipose tissue to maintain volume is an important issue when regenerating large-sized adipose tissue for plastic surgery. For this reason, we mixed the heart dECM gel containing angiogenic factors with adipose dECM gel. In this study, the effect of angiogenesis was compared with that of the adipose dECM alone, and experiments were conducted to determine the ratio of optimized heart dECMs to adipose dECMs.

As shown in Figure 4, more blood vessels were induced in the heart dECM mixed group than in the adipose dECM alone group. In addition, the higher the ratio of heart dECMs, the more vWF^+^ and α-SMA^+^ cells were observed. In numerous studies, heart dECMs have been used to treat cardiac diseases or enhance angiogenesis during tissue regeneration. We speculated that the heart dECM improved angiogenesis because of angiogenic factors in the hydrogel. According to the microarray analysis, heart dECMs contained various angiogenic factors not contained in adipose dECMs, such as the FGF family, PDGF, and angiopoietin. The FGF family promotes the recruitment of mesenchymal cells to the vessel wall, an essential mechanism for the muscularization of nascent capillaries [33]. Moreover, PDGF is essential for vascular stabilization by the recruitment of mesenchymal progenitors. A lack of PDGF leads to fragile neovasculature, which is typical of pathological angiogenesis. Ji Hyun Kim et al. reported that dual delivery of PDGF-bb and FGF-2 enhanced angiogenesis and retained mature blood vessels [31].

Additionally, Pieper et al. reported that collagen–heparan sulfate matrices with bFGF promoted angiogenesis and tissue regeneration. Heparan sulfate binds bFGF and enhances angiogenesis in vivo [34]. Additionally, Sonya B et al. reported that the GAG component contained in the dECM provided the moiety for a heparin-binding growth factor and enhanced neovascularization through the retention and delivery of a heparin-binding growth factor [35]. Therefore, the results of this study suggest that the angiogenesis-inducing growth factors contained in heart dECMs enhanced vascularization and maximized the effect by providing a moiety capable of binding these growth factors.

Figure 6 shows the histological morphology of the samples harvested 12 weeks after subcutaneous implantation in nude mice. Mature adipocytes were found in the groups containing dECM hydrogel and hADSCs. On the other hand, immature cells were observed only in the preadipocyte seeded group because it was difficult to receive any signals by host tissues or the dECM. Groups 1 to 3 contained adipose tissue-derived dECM hydrogels. According to the microarray analysis, adipogenic factors, such as IGF, IGF-BP, and lipocalin remained in the adECM hydrogel after decellularization. We performed a real-time PCR analysis to confirm that these adipogenic factors differentiated hADSCs into adipocytes. As a result, it was confirmed that the adipogenic pathway containing PPAR-γ, c/ebp, and LPL was activated, and the level of gene expression increased as the ratio of adipose dECM hydrogels increased. Falguni Pati et al. reported that various proteins or peptides within the decellularized adipose tissue enhanced the regeneration of adipose tissue [20,22]. They observed a rich source of collagen, laminin, fibronectin, and many other bioactive molecules using a quantitative analysis of extracellular matrices. These components can mimic native tissues and accelerate adipose tissue formation.

The relative quantitative analysis of the cumulative lipids in the constructs was performed using oil red O dye. The cumulative lipid value was highest in the group with the adECM and the hdECM at a ratio of 80/20. The results of the real-time PCR showed better adipogenesis in the group with more adipose dECM hydrogels, but different results were obtained from the in vivo study. This indicates that adipogenesis, cell survival, and tissue maturation through angiogenesis are key issues in the regeneration of mature adipose tissue. Cao et al. reported that neovascularization and adipogenesis are temporally and spatially coupled processes, and they continue to interact via paracrine signaling systems. Activated adipocytes produce multiple angiogenic factors, including leptin and angiopoietins [36]. According to this study, the seeded cells survived due to mass transfer, and the initial angiogenesis improved angiogenesis after adipogenesis. These synergies are thought to be most effective when angiogenesis and adipogenesis are simultaneously enhanced.

In the present study, we attempted to regenerate large-sized tissue by introducing a channel structure inside the scaffold and using adipose and heart dECMs. As a result, the survival of seeded cells and angiogenesis was improved, which enabled the regeneration of mature tissue. However, in terms of lipid accumulation, one of the functions of mature adipose tissue, the constructs developed in this study, contained approximately 30% lipids compared to native adipose tissue. In the real-time PCR data, the initial adipogenesis signaling, PPAR-γ, gradually decreased with time, not only because of the initial signaling but also because of the lack of the sustained signaling of adipogenic factors. To overcome this issue, our future work will include studies to improve adipogenesis, and we plan to apply a strategy for the clinical application of patient-specific large-sized adipose tissue.

## 4. Materials and Methods

### 4.1. The Fabrication of a Poly(Lactide-Co-Caprolactone) Scaffold

A patient-specific elastic scaffold with a channel and pore structure was developed using 3D printing technology and the gel pressing method. To begin, a dome-shaped sacrificial mold containing an inner channel structure was designed using the imaging software SketchUp (Google Inc. Mountain View, CA, USA). It had a diameter of 13 mm, thickness of 6.5 mm, and channel thickness of 800 μm. It was fabricated with polyvinyl alcohol (PVA) using a 3D printer (FDM, Vis Tech Korea, Daejeon, Korea). The PVA mold was printed at 200 °C and the thickness of the layer was 200 μm.

PLCL was synthesized as described previously [23,37]. After PLCL was dissolved in tetrahydrofuran (THF), NaCl particles (300–500 μm) were added to the solution (PLCL: NaCl = 10:90 *w*/*w*%). The PLCL/NaCl paste was prepared by evaporating the THF solvent [23]. The PVA sacrificial mold was filled with PLCL/NaCl paste, and the residual solvent was removed in a vacuum oven for 5 days. Then, the PVA and NaCl particles were removed by salt leaching and sonication for 3 days. Finally, PLCL scaffolds with channel and pore structures were produced after lyophilization.

### 4.2. The Preparation of Decellularized Extracellular Matrix-Based Hydrogels

Adipose tissue was collected from St Mary’s Hospital (Seoul, Korea) after the liposuction of donors between the ages of 30 and 55 with their informed consent and under the approval of the Catholic University of Korea Institutional Review Board. Porcine heart tissues were obtained from a slaughterhouse. The chopped adipose tissue was decellularized in 0.5% SDS solution, with stirring, for 48 h followed by isopropyl alcohol to remove the lipids for 48 h by changing the solutions every 12 h. Then it was washed with PBS and treated with a solution of 0.1% peracetic acid in 4% ethanol for 4 h. Next, the tissue was washed with PBS for 72 h and lyophilized. The chopped heart tissue was decellularized, by stirring, in a 1% SDS solution for 48 h followed by a treatment with 1% Triton X-100 solution for 30 min. The decellularized heart tissue was washed with PBS for 72 h and lyophilized [20].

The dried decellularized tissue was ground into a fine powder with a cryomiller (FreezerMill, SPEX Sample Prep., Metuchen, NJ, USA). The dECM powder was dissolved in 3% acetic acid with 10 mg of pepsin for 48 h (30 mg/mL). Then, a 5N NaOH solution was added to the dECM solution for neutralization [20].

### 4.3. The Characterization of Scaffolds

The morphologies of the scaffolds were examined by scanning electron microscopy (SEM, Hitachi, Tokyo, Japan) operating at 15 kV. Porosity was measured using a mercury intrusion porosimeter (AutoPore IV, Micromeritics, Tokyo, Japan). To investigate the mechanical properties of the scaffolds, samples were compressed until 60% strain (1.5 mm/min, Instron, Norwood, MA, USA) [23].

### 4.4. The Characterization of dECM Hydrogels

#### 4.4.1. Quantification of DNA

The residual DNA of the decellularized tissue was measured using the DNeasy^®^ Blood and Tissue Kit (Qiagen, Germantown, MD, USA). Tissue samples (20 mg) were collected, and DNA was extracted using a tissue lysis buffer. The concentration of ng DNA/mg tissue was analyzed using a NanoDrop (Daemyung Science, ND-1000, Seoul, Korea) in samples.

#### 4.4.2. Quantification of Collagen and Glycosaminoglycan (GAG)

Collagen content was measured using an assay kit (Sircol Collagen Assay, Biocolor Life Science, Northern Ireland, UK). Briefly, collagen was extracted by cold acid-pepsin (pepsin concentration, 0.1 mg/mL in 0.5 M acetic acid) at 4 °C. After treatment with the dye reagent, optical density was measured using a microplate reader at 525 nm wavelength.

Glycosaminoglycan (GAG) levels in the constructs were determined using a dimethylmethylene blue (DMB) assay. Briefly, the constructs were homogenized and digested in 1 mL of papain solution containing 125 µg/mL papain (Sigma, St. Louis, MO, USA) and 100 mM EDTA (Sigma) for 18 h at 60 °C. GAG concentrations were estimated by treating the samples with the DMB dye (Sigma) [24].

#### 4.4.3. Microarrays for Determination of Adipogenic and Angiogenic Factors

Cytokines for adipogenesis and angiogenesis in the adipose and heart dECMs were investigated using a microarray kit (Proteome Profiler Array, R&D Systems, Minneapolis, MN, USA). Native and decellularized tissue samples were treated with 1% (*v*/*v*) Triton X-100 (Biosesang, Seongnam-si, Korea) in PBS with protease inhibitors and chopped for digestion. After homogenization, freezing, thawing, and centrifugation for 5 min to remove cell debris, supernatants containing cytokines were added to each array membrane on a shaker overnight at 4 °C. After treatment with the detection antibody, a Chemi Reagent Mix was added to the membranes. Finally, the membranes were exposed using an X-ray imaging machine (RAS-3000, Fuji, Aichi, Japan).

### 4.5. In Vitro Adipogenic Gene Expression by a Real Time Polymerase Chain Reaction (PCR)

The gene expression of the constructs was examined during in vitro differentiation. The adipogenesis effects of the PLCL scaffold/dECM hydrogel constructs were investigated using hADSCs in vitro. Human subcutaneous adipose tissue samples were obtained from the abdomens of seven female donors between 35 and 54 years of age with the approval of the Catholic University of Korea Institutional Review Board. The tissues were processed to isolate hADSCs, as described previously. hADSCs obtained from different patients were expanded between passages two and three and were randomly used in the experiments.

To prepare the scaffold/dECM hydrogel/cell constructs, cultured hADSCs were collected by trypsin (Corning Cellgro, Manassas, VA, USA) treatment and then resuspended in a culture medium (CM). The suspension of hADSCs (3.5 × 10^6^ cells/scaffold) was dispersed in the dECM hydrogel and then the cell/hydrogel suspension was seeded into the PLCL scaffolds. Next, the constructs were incubated in the well plate at 37 °C and 5% CO_2_ for 4 h and then filled with a culture or conditioned media. The CM was used for the negative control and the experimental groups, and the adipogenic medium (AM) was used for the positive control. The constructs were cultured in each medium for 7, 14, and 21 days and were then collected for RNA isolation (Group 1: scaffold + hADSCs in CM; Group 2: scaffold + adipose dECM hydrogel + hADSCs in CM; Group 3: scaffold + adipose dECM/heart dECM (80/20) + hADSCs in CM; Group 4: scaffold + adipose dECM/heart dECM (50/50) + hADSCs in CM; Group 5: scaffold + hADSCs in AM).

Total RNA was extracted using the RNeasy Mini Kit (Qiagen, Hilden, Germany) according to the manufacturer’s protocol. Subsequently, 2 µg of RNA was reverse-transcribed into cDNA in a 20 μL reaction using an Omniscript System (Qiagen, Hilden, Germany). A real-time polymerase chain reaction (PCR) was performed using an Applied Biosystems 7500 system (Applied Biosystems, Foster City, CA, USA) in conjunction with the Power SYBR Green PCR Master Mix (Applied Biosystems). The real-time PCR was conducted in a final volume of 25 μL using 2–2.5 μL of cDNA as the template [24]. The oligonucleotide primers used are listed in Table 1.

### 4.6. In Vivo Studies of Scaffold/Hydrogel/hADSC Constructs

Vascularized adipose tissue regeneration was investigated in PLCL scaffolds/dECMs/hADSCs in vivo. The samples were prepared in the same way as in the in vitro differentiation tests. These constructs were subcutaneously implanted in immune-deficient nude mice after incubation for 4 h (Group 1: scaffold + adipose dECM + hADSCs; Group 2: scaffold + adipose dECM/heart dECM (80/20) + hADSCs; Group 3: scaffold + adipose dECM/heart dECM (50/50) + hADSCs; Group 4: scaffold + preadipocytes). The constructs were harvested at 1, 4, and 12 weeks after implantation.

#### 4.6.1. Histological and Immunofluorescence Analysis

For histological analyses, the constructs were fixed in 10% *v*/*v* buffered formalin, dehydrated in a graded ethanol series, and embedded in paraffin. The sampled tissues (*n* = 3) were sectioned at a thickness of 6 µm and then stained with hematoxylin and eosin (H&E) staining. The sections were examined under a light microscope (Nikon, Tokyo, Japan) [38].

To evaluate the density of vascular cells in the scaffolds 1 and 4 weeks after implantation, endothelial cells and vascular smooth muscle cells were stained with a polyclonal rabbit anti-human von Willebrand factor antibody (vWF, 1:100, Abcam, Waltham, MA, USA) and a monoclonal mouse anti-human α-smooth muscle actin antibody (α-SMA, 1:100, Santa Cruz Biotech, Dallas, TX, USA), respectively. vWF- and α-SMA-positive vessels in three random fields were evaluated, and vWF^+^ cell densities and α-SMA^+^ vessel densities were quantified using the image analysis program ImageJ (*n* = 3 per group). The maturation index was quantified as the ratio of α-SMA-positive vessels to the total number of vessels [38].

Adipogenic markers (type IV collagen and lipoprotein lipase, Abcam, Waltham, MA, USA) secreted by the seeded cells were detected by immunofluorescence staining. Nuclei were counterstained with 40,6-diamidino-2-phenylindole (Molecular Probes, Eugene, OR, USA) [22].

The apoptosis of seeded cells was detected using the DeadEndTM Rluoromeric TUNEL System (Promega, Madison, WI, USA) 1, 4, and 12 weeks after implantation. The number of TUNEL-positive nuclei and the total number of nuclei in five different fields were counted. The TUNEL+ cell density (%) is expressed as the ratio of TUNEL-positive nuclei to the total number of nuclei [39].

#### 4.6.2. The Relative Quantification of Accumulative Lipids by Oil Red O Dye

Samples that were transplanted into nude mice and harvested after 12 weeks were fixed in 10% (*v*/*v*) buffered neutral formalin for 24 h. Then, 50 mg of each sample was chopped and gently rinsed with PBS. The oil red O solution (500 μL) was added to each sample and incubated for 15 min at room temperature with shaking. Samples were washed with distilled water three times for 10 min each time with shaking. The oil red O dye that bound to the lipids was eluted by the addition of 1 mL of isopropanol. The optical densities of the samples were measured using a microplate reader at 490 and 570 nm.

### 4.7. Statistical Analyses

All samples were assayed at least in triplicate, and the results are expressed as the standard deviations above and below the mean. All statistical analyses were performed using an ANOVA test (GraphPad Prism 6, GraphPad Software Inc., San Diego, CA, USA). Results were considered statistically significant when the *p*-value was less than 0.05.

## 5. Conclusions

In this study, we developed patient-specific elastic scaffolds and a combination of adECM and hdECM hydrogels for the regeneration of large-sized adipose tissue. We attempted to regenerate large-sized tissue by introducing the channel structure inside the scaffold and using adipose and heart dECMs. A dome-shaped elastic PLCL scaffold with a channel and pore structure had mechanical properties similar to those of native adipose tissue and a highly porous structure. Additionally, the adECM and hdECM hydrogels were used for adipogenesis and angiogenesis, respectively. As a result of the in vivo experiments to optimize the ratio of the two hydrogels, angiogenesis, apoptosis, and adipose tissue formation were the best at the 80/20 ratio of the adECM and the hdECM. Consequently, the results of this study suggest that our patient-specific elastic scaffolds/hydrogel mixtures/cell systems could be a good treatment modality to regenerate large patient-specific adipose tissues.

## Figures and Tables

**Table 1:** List of primers used in the real-time PCR analysis of in vitro samples.

Primer Name	Forward Sequence	Reverse Sequence	Product Size (bp)
hPPAR-γ	TTCAGAAATGCCTTGCAGTG	CCAACAGCTTCTCCTTCTCG	599
hC/EBP-α	CAGAGGGACCGGAGTTATGA	TTCACATTGCACAAGGCACT	107
hLPL	GTCCGTGGCTACCTGTCATT	TGGCACCCAACTCTCATACA	94
hGAPDH	ACAGTCAGCCGCATCTTCTT	ACGACCAAATCCGTTGACTC	94

## Data Availability

All data reported in the manuscript and in the supplementary information.

## Supplementary Materials

The following are available online at https://www.mdpi.com/article/10.3390/ijms222212560/s1.

## References

[1] Brayfield C.A., Marra K.G., Rubin J.P. (2010). Adipose Tissue Regeneration. Curr. Stem Cell Res. Ther..

[2] Casadei A., Epis R., Ferroni L., Tocco I., Gardin C., Bressan E., Sivolella S., Vindigni V., Pinton P., Mucci G. (2012). Adipose Tissue Regeneration: A State of the Art. J. Biomed. Biotechnol..

[3] Patrick C.W. (2000). Adipose tissue engineering: The future of breast and soft tissue reconstruction following tumor resection. Semin. Surg. Oncol..

[4] Choi J.H., Gimble J.M., Lee K., Marra K., Rubin J.P., Yoo J.J., Vunjak-Novakovic G., Kaplan D.L. (2010). Adipose Tissue Engineering for Soft Tissue Regeneration. Tissue Eng. Part B Rev..

[5] Patrick C.W. (2001). Tissue engineering strategies for adipose tissue repair. Anat. Rec..

[6] Visscher L.E., Cheng M., Chhaya M., Hintz M.L., Schantz J.T., Tran P., Ung O., Wong C., Hutmacher W.D. (2017). Breast Augmentation and Reconstruction from a Regenerative Medicine Point of View: State of the Art and Future Perspectives. Tissue Eng. Part B Rev..

[7] Tytgat L., Kollert M.R., van Damme L., Thienpont H., Ottevaere H., Duda G.N., Geissler S., Dubruel P., van Vlierberghe S., Qazi T.H. (2020). Evaluation of 3D Printed Gelatin-Based Scaffolds with Varying Pore Size for MSC-Based Adipose Tissue Engineering. Macromol. Biosci..

[8] Colle J., Blondeel P., de Bruyne A., Bochar S., Tytgat L., Vercruysse C., van Vlierberghe S., Dubruel P., Declercq H. (2020). Bioprinting predifferentiated adipose-derived mesenchymal stem cell spheroids with methacrylated gelatin ink for adipose tissue engineering. J. Mater. Sci.: Mater. Med..

[9] Lin K., Zhang D., Macedo M.H., Cui W., Sarmento B., Shen G. (2019). Advanced Collagen-Based Biomaterials for Regenerative Biomedicine. Adv. Funct. Mater..

[10] Tan H., Rubin J.P., Marra K.G. (2010). Injectable in situ forming biodegradable chitosan-hyaluronic acid based hydrogels for adipose tissue regeneration. Organogenesis.

[11] Yao R., Zhang R., Lin F., Luan J. (2012). Injectable cell/hydrogel microspheres induce the formation of fat lobule-like microtissues and vascularized adipose tissue regeneration. Biofabrication.

[12] Cho W.W., Kim B.S., Ahn M., Ryu Y.H., Ha D.H., Kong J.S., Rhie J.W., Cho D.W. (2021). Flexible Adipose-Vascular Tissue Assembly Using Combinational 3D Printing for Volume-Stable Soft Tissue Reconstruction. Adv. Healthc. Mater..

[13] Van Belleghem S., Torres L., Santoro M., Mahadik B., Wolfand A., Kofinas P., Fisher J.P. (2020). Hybrid 3D Printing of Synthetic and Cell-Laden Bioinks for Shape Retaining Soft Tissue Grafts. Adv. Funct. Mater..

[14] Hong S., Sycks D., Chan H.F., Lin S., Lopez G.P., Guilak F., Leong K.W., Zhao X. (2015). 3D Printing: 3D Printing of Highly Stretchable and Tough Hydrogels into Complex, Cellularized Structures (Adv. Mater. 27/2015). Adv. Mater..

[15] Melchels F.P.W., Domingos M.A.N., Klein T.J., Malda J., Bartolo P.J., Hutmacher D.W. (2012). Additive manufacturing of tissues and organs. Prog. Polym. Sci..

[16] Murphy S.V., Atala A. (2014). 3D bioprinting of tissues and organs. Nat. Biotechnol..

[17] Kolesky D.B., Truby R.L., Gladman A.S., Busbee T.A., Homan K.A., Lewis J.A. (2014). 3D bioprinting of vascularized, heterogeneous cell-laden tissue constructs. Adv. Mater..

[18] Levato R., Visser J., Planell J.A., Engel E., Malda J., Mateos-Timoneda M.A. (2014). Biofabrication of tissue constructs by 3D bioprinting of cell-laden microcarriers. Biofabrication.

[19] Wu Z., Su X., Xu Y., Kong B., Sun W., Mi S. (2016). Bioprinting three-dimensional cell-laden tissue constructs with controllable degradation. Sci. Rep..

[20] Pati F., Jang J., Ha D.-H., Kim S.W., Rhie J.-W., Shim J.-H., Kim D.-H., Cho D.-W. (2014). Printing three-dimensional tissue analogues with decellularized extracellular matrix bioink. Nat. Commun..

[21] Kang H.-W., Lee S.J., Ko I.K., Kengla C., Yoo J.J., Atala A. (2016). A 3D bioprinting system to produce human-scale tissue constructs with structural integrity. Nat. Biotechnol..

[22] Pati F., Ha D.-H., Jang J., Han H.H., Rhie J.-W., Cho D.-W. (2015). Biomimetic 3D tissue printing for soft tissue regeneration. Biomaterials.

[23] Kim S.H., Jung Y., Kim S.H. (2013). A biocompatible tissue scaffold produced by supercritical fluid processing for cartilage tissue engineering. Tissue Eng. Part C Methods.

[24] Kim S.H., Kim S.H., Jung Y. (2015). TGF-β3 encapsulated PLCL scaffold by a supercritical CO_2_-HFIP co-solvent system for cartilage tissue engineering. J. Control. Release.

[25] Kim N., Chung J.J., Jung Y., Kim S.H. (2019). The effect of Substance P/Heparin conjugated PLCL polymer coating of bioinert ePTFE vascular grafts on the recruitment of both ECs and SMCs for accelerated regeneration. Sci. Rep..

[26] Van Houten E.E., Doyley M.M., Kennedy F.E., Weaver J.B., Paulsen K.D. (2003). Initial *in vivo* experience with steady-state subzone-based MR elastography of the human breast. J. Magn. Reason. Imaging.

[27] Velasco M.A., Narváez-Tovar C.A., Garzón-Alvarado D.A. (2015). Design, Materials, and Mechanobiology of Biodegradable Scaffolds for Bone Tissue Engineering. BioMed Res. Int..

[28] Durham E.R., Ingham E., Russell S.J. (2013). Technique for internal channelling of hydroentangled nonwoven scaffolds to enhance cell penetration. J. Biomater. Appl..

[29] Jeong S.I., Kim B.-S., Kang S.W., Kwon J.H., Lee Y.M., Kim S.H., Kim Y.H. (2004). In vivo biocompatibilty and degradation behavior of elastic poly(l-lactide-*co*-ε-caprolactone) scaffolds. Biomaterials.

[30] Zada M.H., Kumar A., Elmalak O., Markovitz E., Icekson R., Domb A.J. (2020). In vitro and in vivo degradation behavior and the long-term performance of biodegradable PLCL balloon implants. Int. J. Pharm..

[31] Kim J.H., Jung Y., Kim S.-H., Sun K., Choi J., Kim H.C., Park Y., Kim S.H. (2011). The enhancement of mature vessel formation and cardiac function in infarcted hearts using dual growth factor delivery with self-assembling peptides. Biomaterials.

[32] Madeddu P. (2005). Therapeutic angiogenesis and vasculogenesis for tissue regeneration. Exp. Physiol..

[33] Wang R.M., Christman K.L. (2016). Decellularized myocardial matrix hydrogels: In basic research and preclinical studies. Adv. Drug Deliv. Rev..

[34] Pieper J.S., Hafmans T., van Wachem P.B., van Luyn M.J.A., Brouwer L.A., Veerkamp J.H., van Kuppevelt T.H. (2002). Loading of collagen-heparan sulfate matrices with bFGF promotes angiogenesis and tissue generation in rats. J. Biomed. Mater. Res..

[35] Seif-Naraghi S.B., Horn D., Schup-Magoffin P.J., Christman K.L. (2012). Injectable extracellular matrix derived hydrogel provides a platform for enhanced retention and delivery of a heparin-binding growth factor. Acta Biomater..

[36] Cao Y. (2007). Angiogenesis modulates adipogenesis and obesity. J. Clin. Investig..

[37] Kim D., Park D., Kim T.H., Chung J.J., Jung Y., Kim S.H. (2021). Substance P/Heparin-Conjugated PLCL Mitigate Acute Gliosis on Neural Implants and Improve Neuronal Regeneration via Recruitment of Neural Stem Cells. Adv. Healthc. Mater..

[38] Kim S.H., Kim J.E., Kim S.H., Jung Y. (2017). Substance P/dexamethasone-encapsulated PLGA scaffold fabricated using supercritical fluid process for calvarial bone regeneration. J. Tissue Eng. Regen Med..

[39] Kim J.H., Jung Y., Kim B.-S., Kim S.H. (2013). Stem cell recruitment and angiogenesis of neuropeptide substance P coupled with self-assembling peptide nanofiber in a mouse hind limb ischemia model. Biomaterials.

