# Supplementary material for: The Regeneration of Large-Sized and Vascularized Adipose Tissue Using a Tailored Elastic Scaffold and dECM Hydrogels
Source: Int J Mol Sci. 2021 Nov 22;22(22):12560. doi: 10.3390/ijms222212560 (PMC8624932; doi:10.3390/ijms222212560)
Supplement: Supplementary file 1 [file ijms-22-12560-s001.zip › ijms-1463307-supplementary.pdf]

Supplementary Material

The Regeneration of Large-Sized and Vascularized Adipose Tissue Using a Tailored Elastic Scaffold and dECM Hydrogels

Su Hee Kim [1,2,†], Donghak Kim [1,3,†], Misun Cha [2], Soo Hyun Kim [1,3,4,*] and Youngmee Jung [1,5,*]

[1] Biomaterials Research Center, Korea Institute of Science and Technology (KIST), 5, Hwarang-ro 14-gil, Seongbuk-gu, Seoul 02792, Korea; sweess@imedifab.com (S.H.K.); cherish7693@korea.ac.kr (D.K.)

[2] R&D Center, Medifab Co. Ltd., 70 Dusan-ro, Geumcheon-gu, Seoul 08584, Korea; cmsbest@imedifab.com

[3] KU-KIST Graduate School of Converging Science and Technology, Korea University, 145 Anam-ro, Seongbuk-gu, Seoul 02841, Korea

[4] Korea Institute of Science and Technology (KIST) Europe, Campus E 7.1, 66123 Saarbrücken, Germany

[5] School of Electrical and Electronic Engineering, YU-KIST Institute, Yonsei University, Seoul 03722, Korea

* Correspondence: soohkim@kist.re.kr (S.H.K.); winnie97@kist.re.kr (Y.J.)

† These authors contributed equally to this paper.

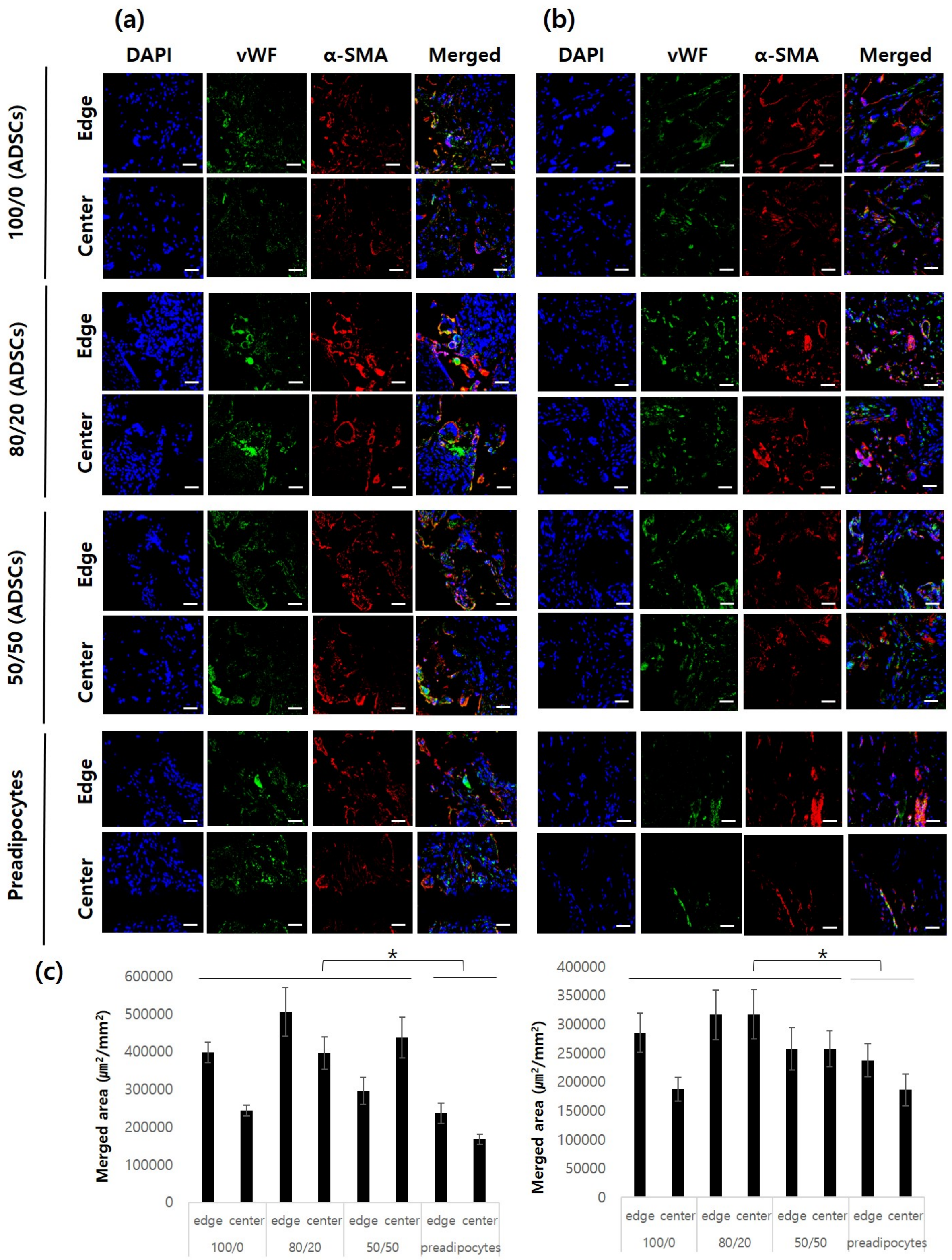

Figure S1. Angiogenesis in constructs. (**a**) Representative immunostaining of endothelial cells (ECs) and smooth muscle cells (SMCs) in each group at 4 and (**b**) 12 weeks after implantation. (**c**) Quantification of the merged area of the vWF⁺ and α-SMA⁺ vessels (μm²/mm²). Scale bar: 100 μm (* $p < 0.05$).

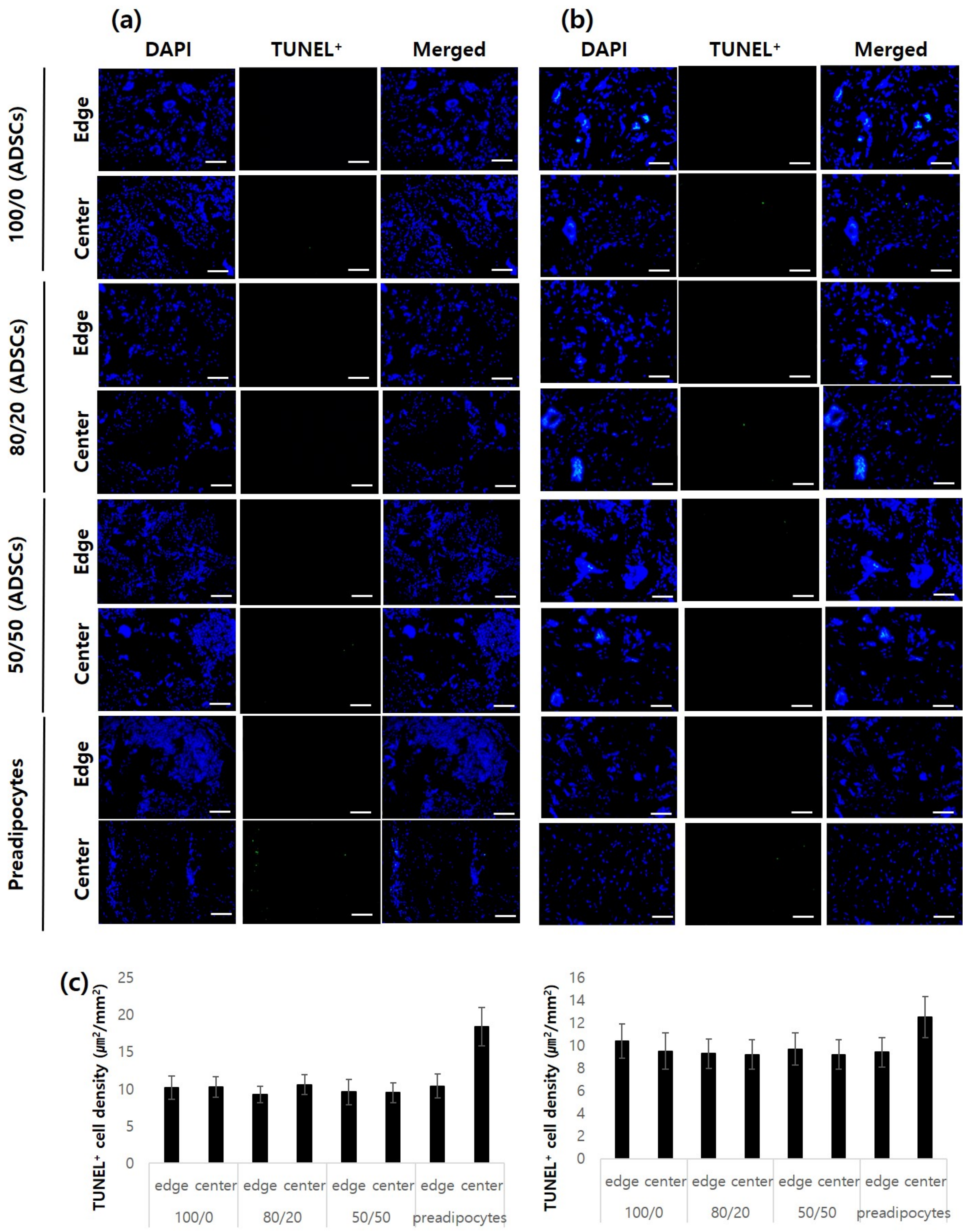

Figure S2. Apoptosis of seeded cells in constructs. (**a**) Representative immunostaining of apoptotic cells in each group at 4 and (**b**) 12 weeks after implantation. (**c**) Quantification of the stained area (μm²/mm²). Scale bar: 100 μm (* $p < 0.05$).
